# Early postoperative pain as a marker of anastomotic leakage in colorectal cancer surgery

**DOI:** 10.1007/s00384-021-03984-w

**Published:** 2021-07-17

**Authors:** Petrus Boström, Johan Svensson, Camilla Brorsson, Martin Rutegård

**Affiliations:** 1 Department of Surgical and Perioperative Sciences, Surgery, Umeå University, Umea, Sweden; 2 Department of Statistics, Umeå School of Business and Economics, Umeå University, Umea, Sweden; 3 Department of Surgical and Perioperative Sciences, Anaesthesiology and Intensive Care Medicine, Umeå University, Umea, Sweden; 4 Wallenberg Centre for Molecular Medicine, Umeå University, Umea, Sweden

**Keywords:** Anastomotic insufficiency, Leak, Dehiscence, Vital sign, Colon, Rectum

## Abstract

**Purpose:**

Even though anastomotic leakage after colorectal surgery is a major clinical problem in need of a timely diagnosis, early indicators of leakage have been insufficiently studied. We therefore conducted a population-based observational study to determine whether the patient’s early postoperative pain is an independent marker of anastomotic leakage.

**Methods:**

By combining the Swedish Colorectal Cancer Registry and the Swedish Perioperative Registry, we retrieved prospectively collected data on 3084 patients who underwent anastomotic colorectal surgery for cancer in 2014–2017. Postoperative pain, measured with the numerical rating scale (NRS), was considered exposure, while anastomotic leakage and reoperation due to leakage were outcomes. We performed logistic regression to evaluate associations, estimating odds ratios (ORs) and 95% confidence intervals (CIs), while multiple imputation was used to handle missing data.

**Results:**

In total, 189 patients suffered from anastomotic leakage, of whom 121 patients also needed a reoperation due to leakage. Moderate or severe postoperative pain (NRS 4–10) was associated with an increased risk of anastomotic leakage (OR 1.69, 95% CI 1.21–2.38), as well as reoperation (OR 2.17, 95% CI 1.41–3.32). Severe pain (NRS 8–10) was more strongly related to leakage (OR 2.38, 95% CI 1.44–3.93). These associations were confirmed in multivariable analyses and when reoperation due to leakage was used as an outcome.

**Conclusion:**

In this population-based retrospective study on prospectively collected data, increased pain in the post-anaesthesia care unit is an independent marker of anastomotic leakage, possibly indicating a need for further diagnostic measures.

**Supplementary information:**

The online version contains supplementary material available at 10.1007/s00384-021-03984-w.

## Introduction

Anastomotic leakage is still a frequent complication after surgery for colorectal cancer [1, 2], with many established risk factors, most of which are known preoperatively, such as sex, body mass index (BMI), American Society of Anesthesiologists’ (ASA) class, tumour height, absence of a diverting stoma and neoadjuvant radiotherapy [3–6]. In addition, certain intraoperative events are known risk factors, including operation duration, level of anastomosis, gross faecal contamination and blood loss [2, 3, 7]. Less is, however, known of very early postoperative predictive factors, where most research has focused on biomarkers in serum, especially C-reactive protein and procalcitonin [8], or in drainage fluid, including inflammatory cytokines [9, 10]. However, the diminishing use of drains, due to lack of evident benefits [11], effectively hinders analyses of drainage fluids, while serum biomarkers seem to be useful only by the third postoperative day [8, 12].

The numeric rating scale (NRS) is the easiest, most frequent and responsive pain variable in clinical practice for describing postoperative pain [13–17]. It has also been associated with a number of complications after surgery, including surgical site infections, ileus, nausea and vomiting, urinary retention and tract infections [18], and for colorectal surgery specifically, length of stay and pulmonary complications [19]. However, these findings were derived from either a single-centre study or an inter-hospital level comparison [18, 19]; so, hitherto, no large, population-based study has been performed, and none has focused on the first postoperative days, when little additional information is available to the clinical team. The purpose of this study was therefore to evaluate the independent predictive ability of early postoperative pain on anastomotic leakage after colorectal cancer surgery.

## Methods

### Inclusion, exclusion and data registries

This is a retrospective, population-based, cohort study, based on prospectively collected data. Patients were identified by cross-referencing the *Swedish Perioperative Registry* (SPOR) with the *Swedish Colorectal Cancer Registry* (SCRCR) and eligible for inclusion if they had undergone any colorectal cancer surgery in which an anastomosis was fashioned in 2014–2017. Exclusion criteria were emergency surgery or direct admission to the intensive care unit after surgery, as well as hospitals with fewer than 50 yearly registrations, or more than 50% missing data on postoperative pain recordings, as we deemed data from such institutions to be of uncertain reliability. This study was approved by the regional ethical review board at Umeå University, Sweden (protocol number: 2018/425–31).

The *SPOR* was created in 2013 and by the end of 2017 had a coverage of 85%, retrieving data from 60 of Sweden’s 90 surgical units [20]. Data is prospectively and automatically collected from perioperative case records. The *SCRCR* was originally created in 1995, with a coverage of at least 97%, and is regularly validated [21]. Data on oncological treatment, surgery and follow-up is entered manually and prospectively. In addition, to find potentially missed cases, the registry is frequently cross-referenced with the National Cancer Registry. Data input is made by standardised forms, including an operative registration form, which is filled out after surgery, usually by the principal surgeon herself. Postoperative variables are entered using a different registration form after 30 postoperative days or at the end of the index admission. All variables were collected from the *SCRCR* except for data on postoperative pain, length of stay at the post-anaesthesia care unit (*PACU)*, and admission to the intensive care unit.

### Exposures and outcome

From the *SPOR,* data was collected on maximal pain experienced by the patient at the *PACU*, as gauged by the staff, using the *NRS,* ranging from 0 to 10, a frequently used and thoroughly validated method of pain documentation [13, 22]. As long as the patient is managed at the *PACU*, every pain registration into the case record is automatically entered into the *SPOR*. Hence, we could retrieve each patient’s single highest pain score recording and use as our exposure variable, whether it was movement-evoked or at rest. The main outcome was anastomotic leakage, which is recorded in the *SCRCR,* given that it is diagnosed within 30 days or during the same admission as the index operation. Additionally, we used reoperation due to leakage as our outcome, in order to include only the clinically most significant anastomotic leaks.

### Statistical analyses

In the primary analyses, pain was categorized dichotomously (NRS 0–3 versus NRS 4–10) [13], and its association with anastomotic leakage was estimated. In our secondary analyses, designed to capture a biological gradient, similar to a dose–response relationship, pain was instead trichotomized as mild (NRS 0–3), moderate (NRS 4–7), or severe (NRS 8–10), in accordance with the nomenclature of earlier research [13, 18]. In addition, pain was investigated as a continuous exposure variable. We performed both univariable and multivariable logistic regression analyses to estimate the association between postoperative pain and anastomotic leakage, using the following established or suspected risk factors for leakage in the multivariable analyses: age at surgery (continuous variable, in years), sex (male/female), ASA score (I, II or III–IV), BMI (< 20, 20–25, 25.01–30 or > 30 kg/m^2^), neoadjuvant therapy (yes/no), clinical tumour stage (I, II, III or IV), tumour site (colon/rectum), intraoperative bleeding (continuous, in ml), operation time (continuous, in minutes), the presence of a defunctioning stoma (yes/no), surgical approach (laparoscopy/laparotomy/converted) and hospital volume (operative procedures per year, in tertiles). Identical analyses were then performed with reoperation due to leakage as outcome.

To account for missing data and minimise bias compared to complete cases analysis, we used multiple imputation with chained Eqs. [23, 24] when conducting our regression analyses, imputing the following variables: postoperative pain, BMI, intraoperative bleeding, clinical tumour stage, operative time and surgical approach. The results were then pooled according to Rubin’s rules [25]. All analyses were also conducted on a complete cases dataset.

The initial surgical approach strongly influences the planned pain treatment, since epidural anaesthesia is preferred in open surgery, while spinal or no regional anaesthesia is more commonly used for laparoscopic procedures. We therefore performed stratified analyses on open, laparoscopic and converted surgery. However, we did not have any data on the actual pain treatment. In addition, due to the difference in incidence and mechanisms behind especially extraperitoneal and intraperitoneal anastomotic leakage, stratification according to tumour site (colon or rectum) was made, where the rectum was defined as the most aboral 15 cm of the large bowel. Subgroup analyses were formally carried out using interaction terms, based on the imputed values of postoperative pain and surgical approach or tumour site, respectively, allowing the estimations to be done using the original multiple imputation, without violating the underlying assumptions of the imputation process. In a sensitivity analysis, the minority of patients who spent more than a full day at the *PACU* were excluded, since a prolonged stay in itself is a strong indicator of a troublesome postoperative course.

Categorical variables were measured as proportions or frequencies. Continuous variables were presented as means and standard deviations, or medians and interquartile ranges if normal distributions could not be assumed. Multilevel mixed-effects logistic regression analyses were performed using odds ratios (ORs) with 95% confidence intervals (CIs), adjusting the standard deviations for clustering of patients within a hospital, since these observations could not be considered independent. Among other things, background characteristics, surgery and pain management tend to be more uniform within than between hospitals. The level of statistical significance was set at 0.05, and all tests were two-tailed. All analyses were made using *Stata 15.1* (StataCorp. 2017. *Stata Statistical Software: Release 15*. College Station, TX: StataCorp LLC.)

## Results

### Background data

**Fig. 1:**
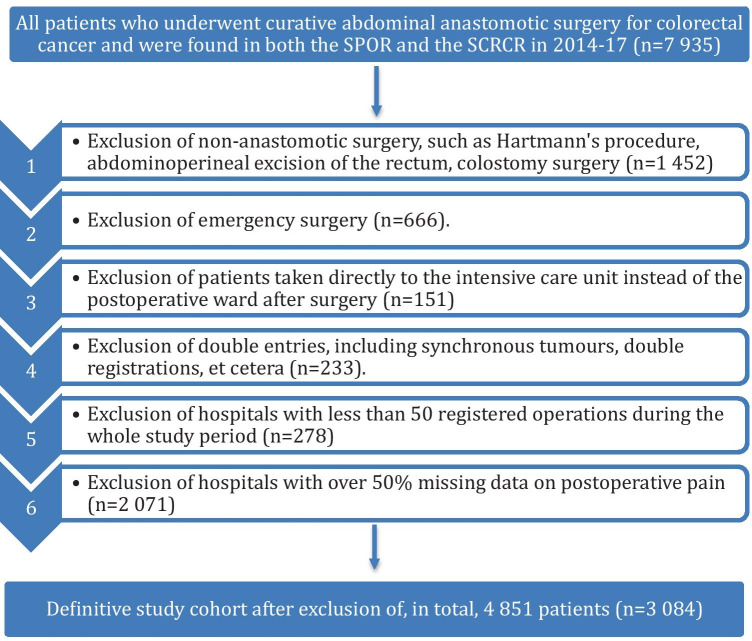
Flowchart depicting the process in which 4851 patients were systematically excluded, yielding a definitive study population of 3084 patients from the initial inclusion of 7935 patients. SPOR, Swedish Perioperative Register; SCRCR, Swedish Colorectal Cancer Registry

A flowchart of the inclusion and exclusion of patients in the study is found in Fig. 1. After exclusion, 3084 patients, who between 1 January 2014 and 31 December 2017, underwent anastomotic colorectal cancer surgery, remained for analyses. Table 1 depicts the background data, stratified by occurrence of anastomotic leakage. The average patient was 72 years old at the time of surgery, had an ASA score of II, a BMI of 25–30, suffered from stage III cancer and had not received any neoadjuvant therapy. The typical surgery was a laparotomy which lasted 196 min on average, with a mean intraoperative bleeding of 159 ml and was performed without a defunctioning stoma.

**Table 1:** Demographic and clinical characteristics by anastomotic leakage for the first imputed dataset in 3084 patients operated for colorectal cancer in Sweden, during 2014–2017, and degree of missingness in the original dataset

Categorical variables		No leakage	Leakage	Missing
		N (%)	N (%)	N (%)
Postoperative pain				528 (17.1)
	NRS 0–3	1 532 (52.9)	76 (40.2)	
	NRS 4–10	1 363 (47.1)	113 (59.8)	
Sex				0 (0)
	Male	1 447 (50.0)	114 (60.3)	
	Female	1 448 (50.0)	75 (39.7)	
ASA score				0 (0)
	I	411 (14.2)	27 (14.3)	
	II	1 618 (55.9)	107 (56.6)	
	III-IV	866 (29.9)	55 (29.1)	
BMI (kg/m^2^)				72 (2.3)
	< 20	159 (5.5)	12 (6.4)	
	20–25	1 109 (38.3)	55 (29.1)	
	25–30	1 095 (37.8)	76 (40.2)	
	> 30	532 (18.4)	46 (24.3)	
Neoadjuvant therapy				0 (0)
	No	2 503 (86.5)	146 (77.3)	
	Yes	392 (13.5)	43 (22.8)	
Tumour site				0 (0)
	Colon	2 253 (77.8)	124 (65.6)	
	Rectum	642 (22.2)	65 (34.4)	
Clinical tumour stage				594 (19.3)
	I	699 (24.2)	32 (16.9)	
	II	695 (24.0)	41 (21.7)	
	III	1 270 (43.9)	93 (49.2)	
	IV	231 (8.0)	23 (12.2)	
Defunctioning stoma				0 (0)
	No	2 332 (80.6)	138 (73.0)	
	Yes	563 (19.5)	51 (27.0)	
Surgical approach				4 (0.1)
	Open	1 765 (61.0)	125 (66.1)	
	Laparoscopy	954 (33.0)	56 (29.6)	
	Converted	176 (6.1)	8 (4.2)	
Hospital volume*				0 (0)
	Low	713 (24.6)	28 (14.8)	
	Medium	1 257 (43.4)	80 (42.3)	
	High	925 (32.0)	81 (42.9)	
Continuous variables		Median (IQR)	Median (IQR)	
Age (years)		73 (65–79)	72 (63–79)	0 (0)
Bleeding (ml)		50 (25–200)	100 (30–300)	46 (1.5)
Operation time (min)		168 (123–232)	185 (140–263)	14 (0.5)

*N* number, *ASA* American Society of Anesthesiologists, *BMI* body mass index, *IQR* interquartile range

*Annual volume of anastomotic colorectal cancer surgery at the operating hospital, divided into tertiles

### Pain, anastomotic leakage and reoperation

**Fig. 2:**
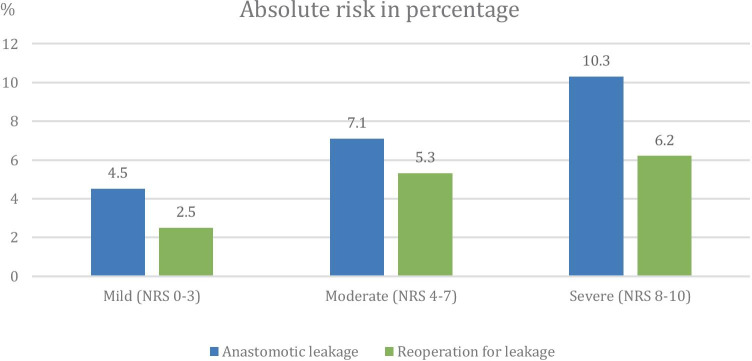
Absolute risk for anastomotic leakage and reoperation due to leakage, stratified by numeric rating scale (NRS) score, on multiple imputation data

A total of 189 (6.1%) patients suffered from anastomotic leakage and 121 (3.9%) patients underwent reoperation due to leakage. All main analyses are displayed in Table 2, which highlights the positive association between exposure and outcome. For all analyses, in both univariable and multivariable settings, pain was associated with an increased risk for anastomotic leakage and reoperation due to leakage. Hence, the association between moderate to severe pain (NRS 4–10) and leakage (OR 1.69, 95% CI 1.21–2.38) or reoperation (OR 2.17, 95% CI 1.41–3.32) remained when adding pertinent covariates to the logistic regression analyses. In addition, the association between pain and leakage was more evident for patients with severe pain (NRS 8–10: OR 2.38, 95% CI 1.44–3.93) than moderate pain (NRS 4–7: OR 1.57, 95% CI 1.07–2.29), with similar results when evaluating reoperation for leakage, as well as in the multivariable analyses. Figure 2 shows the increasing incidence of anastomotic leakage and reoperation with more severe pain.

**Table 2:** Odds ratios (ORs) with 95% confidence intervals (CIs) for the association between pain and anastomotic leakage and reoperation for leakage, respectively, using logistic regression modelling with imputed data

	Anastomotic leakage		Reoperation	
	OR (95% CI)	P value	OR (95% CI)	P value
Univariable				
Pain, dichotomized				
NRS 0–3	1.00 (reference)		1.00 (reference)	
NRS 4–10	1.69 (1.21–2.38)	< 0.01	2.17 (1.41–3.32)	< 0.01
Pain, trichotomized				
NRS 0–3	1.00 (reference)		1.00 (reference)	
NRS 4–7	1.57 (1.07–2.29)	0.02	2.12 (1.35–3.33)	< 0.01
NRS 8–10	2.38 (1.44–3.93)	< 0.01	2.59 (1.40–4.78)	< 0.01
Pain, continuous				
NRS increment*	1.11 (1.05–1.17)	< 0.01	1.13 (1.06–1.20)	< 0.01
Multivariable^a^				
Pain, dichotomized				
NRS 0–3	1.00 (reference)		1.00 (reference)	
NRS 4–10	1.73 (1.22–2.46)	< 0.01	2.13 (1.37–3.30)	< 0.01
Pain, trichotomized				
NRS 0–3	1.00 (reference)		1.00 (reference)	
NRS 4–7	1.62 (1.09–2.39)	0.02	2.07 (1.30–3.30)	< 0.01
NRS 8–10	2.42 (1.43–4.08)	< 0.01	2.61 (1.39–4.91)	< 0.01
Pain, continuous				
NRS increment*	1.11 (1.05–1.17)	< 0.01	1.12 (1.05–1.20)	< 0.01

*OR* odds ratio, *CI* confidence interval, *NRS* numerical rating scale

*NRS increment is defined as one additional full step in NRS, e.g. moving from NRS 4 to NRS 5

^a^The following confounders were controlled for in the multivariable analyses: age, sex, ASA score, BMI, neoadjuvant therapy, clinical tumour stage, tumour site, intraoperative bleeding, operation time, defunctioning stoma, surgical approach and hospital volume

### Stratification analyses

The association between pain and anastomotic leakage, and the need for reoperation, was also seen in the stratified analyses, the full results of which are found in Table 3. Moderate or severe pain (NRS 4–10) remained associated with leakage (OR 1.73, 95% CI 1.12–2.68) when stratifying for surgical approach, whereas the interactions themselves were all insignificant (OR 1.10, 95% CI 0.51–2.39 for laparoscopy and OR 0.55, 95% CI 0.11–2.77 for conversion to open surgery). Similar results followed when using reoperation as outcome. In addition, neither stratification according to tumour site (colon versus rectum) nor exclusion of the 66 patients (2.1%) who spent more than 24 h at *PACU* (data not shown) significantly altered the association between pain and leakage or reoperation. Finally, similar point estimates were found in our complete cases analyses as when multiple imputation was used (Tables 1b, 2b, and 3b in the electronic appendix).

**Table 3:** Odds ratios (ORs) with 95% confidence intervals (CIs) for the association between pain and anastomotic leakage and reoperation for leakage, respectively, for different strata, using logistic regression stratification modelling with imputed data. For NRS 4–10, an OR above one indicates an increased risk of anastomotic leakage and reoperation, respectively, compared to patients with NRS 0–3. The ensuing ORs describe how the odds are modified for the different strata, compared to the reference groups

		Anastomotic leakage		Reoperation	
		OR (95% CI)*	P value	OR (95% CI)*	P value
Surgical approach					
	NRS 4–10	1.73 (1.12–2.68)	0.01	2.31 (1.32–4.07)	< 0.01
	Open	1.00 (reference)		1.00 (reference)	
	Laparoscopy	1.10 (0.51–2.39)	0.81	0.85 (0.33–2.16)	0.73
	Converted	0.55 (0.11–2.77)	0.47	0.47 (0.07–3.18)	0.44
Tumour site					
	NRS 4–10	1.36 (0.76–2.43)	0.30	2.33 (1.00 5.40)	0.05
	Rectum	1.00 (reference)		1.00 (reference)	
	Colon	1.45 (0.70–3.00)	0.32	0.89 (0.34–2.32)	0.81

*OR* odds ratio, *CI* 95% confidence interval, *NRS* numerical rating scale

*The following confounders were controlled for in the multivariable analyses, in addition to the interaction terms: age, sex, ASA score, BMI, neoadjuvant therapy, clinical tumour stage, tumour site, intraoperative bleeding, operation time, defunctioning stoma, surgical approach and hospital volume

## Discussion

### Summary

In this population-based cohort, postoperative pain at the *PACU* was associated with an increased risk of anastomotic leakage and reoperation due to leakage. This association became more pronounced for patients with increasing severity of pain. Pain seems to be a useful early marker for leakage.

### Weaknesses

The main weakness of the current study is the secondary nature of our data. The *SPOR* is a recently established registry, which forced us to use a rather short time period, as the start of the study period was chosen to match the point in time when data were sufficiently robust. Before 2014, the lack of coverage and degree of missing effectively hindered inclusion into our study cohort. Additionally, it is more difficult to ascertain the validity of data in a new registry, though postoperative pain evaluation is emphasized in clinical practice [20]. The registry also lacks reliable data on other anaesthesiologic parameters such as vital signs and analgesic treatment, why we could neither include them in the regression analyses, nor eliminate the possibility that the sought association between pain and leakage is already better predicted by other deranged vital signs. In addition, the *SCRCR* also lacks some data which would be beneficial to this study, such as the timing of diagnosis and details regarding reoperation for anastomotic leakage.

The large degree of missing data on postoperative pain could lead to both bias and inefficiency. We have, however, a priori, excluded hospitals with a large degree of missing data, to increase the reliability of the dataset. In theory, multiple imputation has the potential to yield less biased results than complete case analyses would, especially for categorical outcomes [26], and fits well with this study’s large number of patients and variables. However, the underlying assumption of *missing at random* is indemonstrable and if erroneous could induce bias instead of alleviating it. Yet, the similar results from the imputed and complete datasets suggest limited bias issues.

Measurement bias is a potential pitfall in this kind of study, since increased postoperative pain could prompt investigations, which would render a diagnosis for what would otherwise have been an undetected leakage. However, colorectal surgeons in Sweden almost exclusively register *symptomatic* leakage into the *SCRCR,* as evidenced by a validation study, in which asymptomatic leaks contributed to only 1.7% of all leakage in rectal cancer surgery [27]. Hence, anastomotic leakage in this study should not be the result of radiology alone. Another drawback is our inability to guarantee that the leak was not already evident at the time of the pain measurement, even though very early leaks are rare [28–30]. As we lack data on other vital signs in PACU, as well as other anaesthesiologic variables, we could neither include them in the regression analyses, nor eliminate the possibility that the only thing pain evaluation achieves is to predict leaks, already better predicted by other deranged vital signs.

### Strengths

The major strength of this study is the ability to conduct a population-based cohort study by cross-referencing two national quality registries. In addition, the large number of included patients enabled us to conduct robust multivariable analyses, including stratification analyses with sufficient power, as well as the aforementioned handling of missing data. Finally, the association between exposure and outcome did not depend on how the pain variable was used—continuously, dichotomized or trichotomized.

### Literature review and biological mechanisms

How is the association between postoperative pain and anastomotic leakage to be understood? First of all, increased pain could be a symptom of an already occurring event, as many complications, including the frequent peritonitis due to anastomotic leakage, are indeed painful. Secondly, pain could be an indication for an analgesic treatment, the adverse effect of which are related to complications, such as the possible association between non-steroidal anti-inflammatory drugs and anastomotic leakage [31–33], postoperative ileus and systemic opioid treatment [34] or urinary retention and the need for bladder catheterization in patients with epidural anaesthesia [35]. Finally, and more speculatively, pain could also be part of a causal mechanism which indirectly leads to anastomotic leakage, for example by activation of the sympathetic nerve system and stress hormones [36, 37], which could negatively influence wound healing, including that of the anastomosis [38–41]. Our study was not designed to determine the mechanism, if any, with which pain might be associated to leakage.

Rough et al. showed both in vitro how beta blockade decreased the hyperinflammatory response from surgical trauma and in vivo how mice treated with beta blockers had lower mortality [42]. As hyperactivation of the sympathetic response is associated with cardiovascular incidents during the perioperative period [43], earlier research on beta blockade focused on its ability to decrease cardiovascular complications after non-cardiac surgery [44, 45]. Recently, however, Ahl et al., in a large population-based study on rectal cancer surgery, showed a surprising negative association between preoperative beta blockade use and anastomotic leakage (incidence rate ratio 0.68, 95% CI 0.51–0.91) and mortality (hazard ratio 0.43, 95% CI 0.37–0.52) in a univariable setting, where intuitively the opposite would be expected [46]. These findings could be attributed to the hyperadrenergic state, induced by major surgery and reinforced by pain, both of which increase physiological stress, leading to cardiovascular incidents and hampering tissue healing [43].

Two large, well-conducted studies on the association between pain and postoperative complications are worth mentioning. Regenbogen et al. performed a retrospective study using prospectively collected data on 7221 patients who underwent a colorectal resection and found hospitals with lower pain scores on postoperative day 1 to report fewer complications and readmissions [19]. Our own study design instead used data on individual patients, while correcting for intra-hospital dependence of observations, though the sought association might best be observed on an inter-hospital level. The only large, well-designed study on an individual patient level was conducted by van Boekel et al., including 1014 surgical patients. Both unacceptable pain and maximal movement-evoked pain on early postoperative days were used as predictors. Overall complication rate was 34%, increasing from 25% for NRS 0 to 45% for NRS 10, with a significantly higher risk in patients with unacceptable pain (adjusted OR 2.17, 95% CI 1.51–3.10), which persisted when excluding complications with only Clavien-Dindo scores I–II. These results from a broad surgical population in a single institution are similar to ours, but the proportion of colorectal surgery was not stated and no patient seems to have suffered from anastomotic leakage, even though this is a common complication [18].

## Implications and conclusions

Increased pain on the first postoperative day after colorectal cancer surgery should not only suggest insufficient analgesia but also raise the suspicion for anastomotic leakage. If seen in conjunction with one or more established risk factors or any additional deviation from the normal postoperative course, further diagnostic measures seem indicated. However, additional research with prospective anaesthesiologic data such as vital signs and analgesic management is warranted, to further demarcate the independent predictive abilities of postoperative pain on leakage.

## Supplementary information

Below is the link to the electronic supplementary material.

Supplementary file1 (DOCX 21 KB)
