# Supplementary material for: Early postoperative pain as a marker of anastomotic leakage in colorectal cancer surgery
Source: Int J Colorectal Dis. 2021 Jul 17;36(9):1955–63. doi: 10.1007/s00384-021-03984-w (PMC8346442; doi:10.1007/s00384-021-03984-w)
Supplement: Supplementary file 1 — Supplementary file1 (DOCX 21 KB) [file 384_2021_3984_MOESM1_ESM.docx]

**Table 1b.** Epidemiological and surgical characteristics by anastomotic leakage, for complete cases. Corresponds to Table 1 in the original manuscript.

|  |  | No leakage | Anastomotic leakage |
| --- | --- | --- | --- |
| Categorical variables |  | **N (%)** | **N (%)** |
| Pain |  |  |  |
|  | NRS 0–3 | 1366 (54.3) | 63 (39.9) |
|  | NRS 4–10 | 1149 (45.7) | 95 (60.1) |
| Sex |  |  |  |
|  | Male | 2454 (50.4) | 177 (62.77) |
|  | Female | 2419 (49.6) | 105 (37.2) |
| ASA score |  |  |  |
|  | I | 662 (13.6) | 43 (15.3) |
|  | II | 2676 (55.0) | 139 (53.0) |
|  | III-IV | 1532 (31.5) | 89 (31.7) |
| BMI |  |  |  |
|  | <20 | 276 (5.8) | 13 (4.7) |
|  | 20–25 | 1808 (37.8) | 83 (30.2) |
|  | 25–30 | 1826 (38.2) | 116 (42.2) |
|  | >30 | 870 (18.2) | 63 (22.9) |
| Neoadjuvant therapy |  |  |  |
|  | No | 4193 (86.1) | 216 (76.6) |
|  | Yes | 680 (14.0) | 66 (23.4) |
| Tumour site |  |  |  |
|  | Colon | 3780 (77.6) | 180 (63.8) |
|  | Rectum | 1093 (22.4) | 102 (36.2) |
| Clinical tumour stage |  |  |  |
|  | I | 906 (22.4) | 33 (15.2) |
|  | II | 932 (23.0) | 44 (20.3) |
|  | III | 1846 (45.6) | 114 (52.5) |
|  | IV | 363 (9.0) | 26 (12.0) |
| Defunctioning stoma |  |  |  |
|  | No | 3840 (78.8) | 200 (70.9) |
|  | Yes | 1033 (21.2) | 82 (29.1) |
| Surgical approach |  |  |  |
|  | Open | 2810 (57.8) | 174 (61.7) |
|  | Laparoscopy | 1718 (35.3) | 91 (32.3) |
|  | Converted | 354 (6.9) | 17 (6.0) |
| Hospital volume |  |  |  |
|  | Low | 1599 (32.0) | 76 (27.0) |
|  | Medium | 1485 (30.5) | 90 (31.9) |
|  | High | 1829 (37.5) | 116 (41.1) |
|  |  |  |  |
| Continuous variables |  | **Median (IQR)** | **Median (IQR)** |
|  | Age | 72 (65-79) | 71 (62-78) |
|  | Bleeding | 75 (25-200) | 100 (30-300) |
|  | Operation time | 185 (137-256) | 199 (144-290) |

N = number; ASA = American Society of Anesthesiologists; BMI = Body mass index. Hospital volume = annual volume of anastomotic colorectal cancer surgery at the operating hospital.

**Table 2b.** Odds ratios with 95% confidence intervals for the association between pain and anastomotic leakage and reoperation, respectively, using complete cases. Corresponds to Table 2 in the original manuscript.

| Univariable | Anastomotic leakage |  | Reoperation |  |
| --- | --- | --- | --- | --- |
|  | **OR (95 % CI)** | **p value** | **OR (95 % CI)** | **p value** |
| Pain, dichotomized |  |  |  |  |
| NRS 0–3 | 1.00 (reference) |  | 1.00 (reference) |  |
| NRS 4–10 | 1.72 (1.23-2.40) | <0.01 | 2.22 (1.46-3.36) | <0.01 |
| Pain, trichotomized |  |  |  |  |
| NRS 0–3 | 1.00 (reference) |  | 1.00 (reference) |  |
| NRS 4–7 | 1.52 (1.06-2.18) | 0.02 | 2.10 (1.35-3.27) | <0.01 |
| NRS 8–10 | 2.49 (1.54-3.90) | <0.01 | 2.63 (1.46-4.73) | <0.01 |
| Pain, continuous |  |  |  |  |
| NRS +1 | 1.11 (1.05-1.17) | <0.01 | 1.13 (1.07-1.21) | <0.01 |
| Multivariable | **Anastomotic leakage** |  | **Reoperation** |  |
|  | **OR (95% CI** | **p value** | **OR (95% CI)** | **p value** |
| Pain, dichotomized |  |  |  |  |
| NRS 0–3 | 1.00 (reference) |  | 1.00 (reference) |  |
| NRS 4–10 | 2.07 (1.38-3.12) | <0.01 | 2.50 (1.49-4.19) | <0.01 |
| Pain, trichotomized |  |  |  |  |
| NRS 0–3 | 1.00 (reference) |  | 1.00 (reference) |  |
| NRS 4–7 | 1.89 (1.21-2.94) | <0.01 | 2.42 (1.40-4.16) | <0.01 |
| NRS 8–10 | 2.64 (1.50-4.63) | <0.01 | 2.78 (1.37-5.65) | <0.01 |
| Pain, continuous |  |  |  |  |
| NRS increment* | 1.12 (1.06-1.19) | <0.01 | 1.13 (1.05-1.22) | <0.01 |

OR = odds ratio; CI = 95 % confidence interval; NRS = numerical rating scale.

* NRS increment is defined as one additional step in NRS, e.g. moving from NRS 4 to NRS 5.

**Table 3b.** Stratification analyses using a dichotomized pain score and interaction terms between pain and the stratas of interest, and sensitivity analyses with only patients who spent less 24 hours in the postoperative ward, using complete cases. Corresponds to Table 3 in the original manuscript.

|  |  | Anastomotic leakage |  | Reoperation |  |
| --- | --- | --- | --- | --- | --- |
| Surgical approach |  | **OR (95% CI)** | **p value** | **OR (95% CI)** | **p value** |
|  | NRS 4–10 | 1.65 (0.98-2.76) | 0.06 | 2.07 (1.06-4.05) | 0.03 |
|  | Open | 1.00 (ref) |  | 1.00 (ref) |  |
|  | Laparoscopy | 1.81 (0.68-4.81) | 0.24 | 1.59 (0.47-5.33) | 0.46 |
|  | Converted | 1.02 (0.08-12.40) | 0.99 | 0.44 (0.025-7.90) | 0.58 |
| Tumour site |  |  |  |  |  |
|  | NRS 4–10 | 1.85 (1.00-3.44) | 0.05 | 3.47 (1.32-9.11) | 0.01 |
|  | Rectum | 1.00 (ref) |  | 1.00 (ref) |  |
|  | Colon | 1.21 (0.54-2.73) | 0.64 | 0.63 (0.21-1.94) | 0.42 |
| Posoperative stay <24 h |  |  |  |  |  |
|  | NRS 0–3 | 1.00 (ref) |  | 1.00 (ref) |  |
|  | NRS 4–10 | 1.93 (1.25-2.98) | <0.01 | 2.25 (1.30-3.90) | <0.01 |

OR = odds ratio; CI = 95 % confidence interval; NRS = numerical rating scale; ref = reference value
